# Case Report: A rare complication of non-hypertensive HELLP syndrome—microangiopathic coagulation activation leading to postpartum DIC with acute kidney injury

**DOI:** 10.3389/fmed.2025.1641271

**Published:** 2025-10-31

**Authors:** Chunping Li, Jianping Zhang, Yong Tang

**Affiliations:** Sichuan Jinxin Xinan Women and Children’s Hospital, Chengdu, Sichuan, China

**Keywords:** HELLP syndrome, disseminated intravascular coagulation, thrombotic microangiopathy, acute kidney injury, fluid therapy, obstetric coagulation disorders, case report

## Abstract

Thrombotic disseminated intravascular coagulation (DIC) associated with hemolysis, elevated liver enzymes, and low platelets (HELLP) syndrome in the absence of typical hypertension is rare, and its pathogenesis differs significantly from that of hemorrhagic DIC. Although the overall incidence is low, disease progression is often rapid and carries a high risk of multi-organ failure. Pregnancy-specific physiologic changes, including endothelial injury, hypercoagulability, and a predisposition to microcirculatory thrombosis, markedly increase the likelihood of microangiopathic coagulation activation in non-hypertensive HELLP patients. Therefore, prompt recognition of coagulation abnormalities and timely, targeted interventions are crucial for improving patient outcomes. Here, we report a case of HELLP syndrome without prenatal hypertension that rapidly progressed to DIC and acute kidney injury (AKI), despite only moderate blood loss (800 mL). This case highlights potential mechanisms underlying microangiopathic coagulation activation and provides an important reference for the clinical recognition and management of such occult coagulopathies.

## 1 Introduction

Obstetric coagulation dysfunction remains a major contributor to perinatal mortality, with classic precipitating factors such as postpartum hemorrhage, placental abruption, and preeclampsia (1). Although DIC has traditionally been linked to massive blood loss, substantial gaps remain in understanding the pathophysiology and management of non-hemorrhagic coagulopathy (2, 3). As a cardinal manifestation of gestational thrombotic microangiopathy (TMA), HELLP syndrome may trigger DIC through endothelial injury and microthrombus formation. However, its clinical course often progresses insidiously, making it prone to misdiagnosis (4). This diagnostic challenge is especially evident in patients without typical hypertensive features (5). Emerging evidence indicates that approximately 30% of HELLP-associated DIC cases involve blood loss of <1,000 mL, despite coagulation factor consumption exceeding anticipated levels (6). However, the dissociation between coagulation deterioration and actual blood loss in these patients remains poorly characterized. Current obstetric DIC management algorithms still revolve around a “massive hemorrhage” paradigm, with no tailored recommendations for microangiopathic coagulopathy. Here, we present a rare case of atypical HELLP syndrome with severe complications, providing clinical insights into this underrecognized condition.

## 2 Case presentation

A 42-year-old woman, height 163 cm, weight 87 kg, G2P1, was admitted on November 15 with “40 weeks of gestation and occasional lower abdominal tightness.” Her past medical history and antenatal examinations were unremarkable. The admission diagnosis was “G2P1 at 40 weeks, intrauterine pregnancy with a LOA singleton viable, and threatened labor.”

### 2.1 Perioperative course

On November 15, the patient underwent emergency cesarean section because fetal heart rate monitoring indicated prolonged deceleration and grade III amniotic fluid contamination. A live male infant was delivered at 10:23 under epidural anesthesia. At 10:53, blood loss reached 600 mL with extensive wound surface oozing, prompting coagulation studies. Laboratory results showed a platelet count of 91 × 10^9^/L and fibrinogen (FIB) of 2.8 g/L, after which 1 g of tranexamic acid was administered intravenously. Coagulation function deteriorated rapidly within 1 h postpartum: at 11:14, blood loss reached 800 mL, FIB dropped to <0.25 g/L, and D-dimer exceeded 8.0 μg/mL. By 12:23, blood loss had increased to 2,000 mL. Despite fluid resuscitation with 2,500 mL of crystalloids and 500 mL of colloids, coagulation function showed no improvement. Metabolic acidosis was observed (pH: 7.24, BE: −8.4, lactate: 2.4).

**Figure 1:**
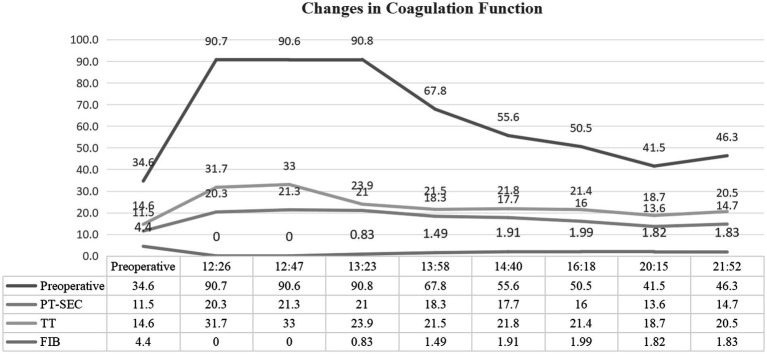
Serial coagulation parameters postpartum. Time-course measurements of coagulation function following emergency cesarean section. Key abnormalities include fibrinogen (FIB) decline from 2.8 g/L to <0.25 g/L (normal: 2–4 g/L), prolonged prothrombin time (PT), and activated partial thromboplastin time (APTT).

**Figure 2:**
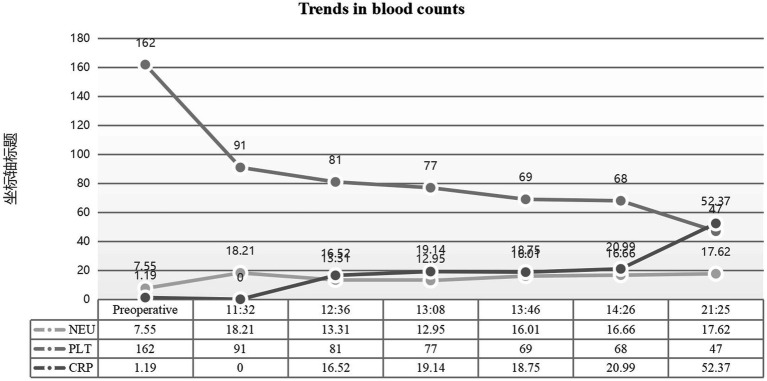
Hematological parameters during disease progression. Dynamic changes in complete blood count. Critical findings include platelet count drop from preoperative 162 × 10⁹/L to 81 × 10⁹/L (normal: 125–350 × 10⁹/L), with subsequent decline to 66 × 10⁹/L, and hemoglobin reduction to 73 g/L post-resuscitation. Neutrophil ratio peaked at 94.9% (normal: 40–75%).

**Figure 3:**
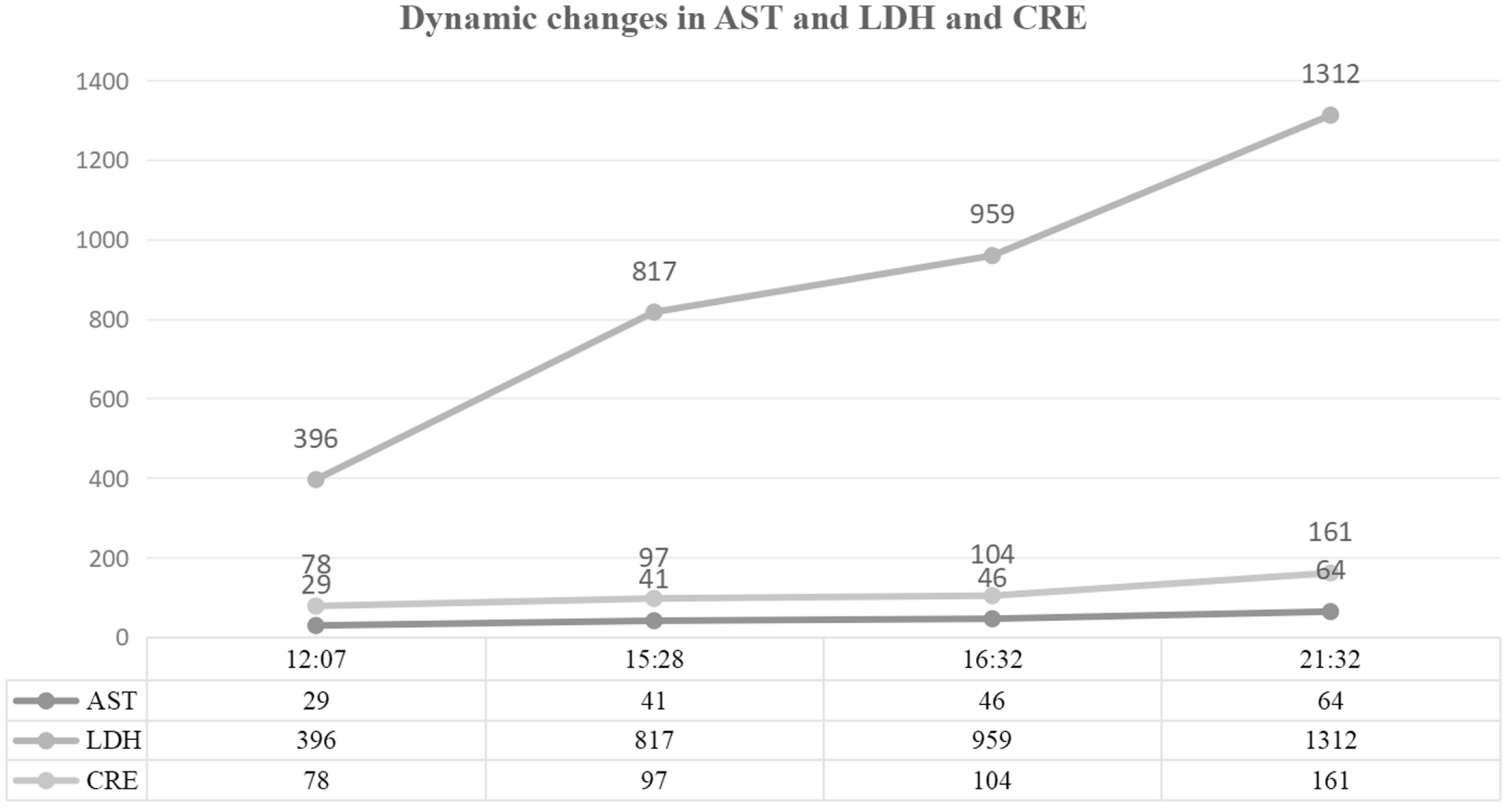
Biochemical markers of organ dysfunction. Evolution of hepatic, renal, and inflammatory indices. Confirmed HELLP syndrome with lactate dehydrogenase (LDH) surge to 1,312 IU/L (normal: 120–250 IU/L) and aspartate aminotransferase (AST) rise to 64 IU/L (normal: 8–33 IU/L). Acute kidney injury evidenced by serum creatinine increase from 55 μmol/L to 161 μmol/L (normal: 45–84 μmol/L).

Multiorgan dysfunction progressed as follows: oliguria developed at 12:07, serum creatinine increased from a baseline of 55 μmol/L to 97 μmol/L at 15:28, and peaked at 161 μmol/L at 21:32. Laboratory findings confirmed HELLP syndrome: lactate dehydrogenase (LDH) rose from a preoperative level of 188 IU/L to 1,312 IU/L, aspartate aminotransferase (AST) increased from 19 IU/L to 64 IU/L, and the platelet count declined sharply from 162 × 10^9^/L to 81 × 10^9^/L. Detailed laboratory results are shown in Figures 1–3.

### 2.2 Multidisciplinary intervention strategy

The surgery was completed at 15:10. For coagulation management, staged transfusion was performed, including 8.5 g of fibrinogen, 1,250 mL of fresh frozen plasma, 10 U of cryoprecipitate, and 8.5 U of packed red blood cells. Additionally, 3 g of tranexamic acid was administered. For circulatory support, volume resuscitation included 5,791 mL of crystalloid solution and 500 mL of colloid solution. Methoxamine was administered in divided doses to maintain a mean arterial pressure above 65 mmHg. Furosemide (60 mg) was administered intermittently for renal protection. Seven hours after the surgery, the patient was transferred to a tertiary care hospital in Chengdu, where continuous renal replacement therapy (CRRT) and additional treatments were initiated. A detailed chronology of the key clinical events and interventions is summarized in Table 1.”

**Table 1:** Timeline of key clinical events and interventions.

Time	Event	Interventions
10:23	Emergency cesarean section commenced	Epidural anesthesia *in situ*
10:27	Delivery of a live male infant	—
10:53	Significant surgical oozing, blood loss 600 mL	First dose IV tranexamic acid (1 g)
11:14	Coagulopathy evident; blood loss 800 mL	Coagulation tests pending
12:07	Onset of oliguria	Fluid status assessment and preparation for diuretic therapy
12:23	Major hemorrhage; blood loss 2,000 mL	Aggressive fluid resuscitation (crystalloids &colloids)
12:30	Hemodynamic instability	Vasopressor support (methoxamine) to maintain MAP >65 mmHg
15:10	End of surgery	Massive transfusion protocol initiated: fibrinogen, FFP, cryoprecipitate, RBCs, additional TXA
~17:10	Preparation for transfer post-stabilization	Arranged transfer to tertiary care center
Tertiary care center	Diagnosis of HELLP syndrome with DIC and AKI	Initiated CRRT and plasma exchange

### 2.3 Diagnostic evolution and regression

At the tertiary hospital, the patient was diagnosed with (i) DIC; (ii) HELLP syndrome; (iii) AKI stage 2; (iv) chronic kidney disease (CKD) stage 2. After 7 days of CRRT and plasma exchange, serum creatinine decreased to 97 μmol/L. However, renal function did not fully recover, and the patient was discharged after providing informed consent. Bone marrow pathology indicated suppression of erythroid hematopoiesis, consistent with TMA changes.

## 3 Discussion

In this patient, DIC driven by coagulation factor consumption was evident before blood loss reached the threshold for obstetric massive hemorrhage. This observation aligns with the pathological hallmark of HELLP syndrome, in which the rate of microthrombus formation markedly exceeds the pace of overt bleeding (7). A significant discordance was observed between the severity of DIC and the actual bleeding volume. The underlying mechanism centers on microangiopathic coagulation activation, characterized predominantly by rapid consumption of coagulation factors. Notably, FIB declined by more than 94% within 1 h—far surpassing the degree expected from hemodilution alone. In conjunction with markedly elevated D-dimer levels (>8.0 μg/mL), indicative of hyperfibrinolysis, and LDH levels exceeding 600 U/L, consistent with microangiopathic hemolysis, these findings support the notion that microthrombosis and fibrinolytic activation act synergistically to accelerate coagulation factor depletion. This mechanism corresponds closely to the pathological model of HELLP-associated TMA proposed by Gunawan et al. (4). A distinctive feature of HELLP-associated DIC is that endothelial injury triggers tissue factor release, thereby activating the extrinsic coagulation pathways. This process results in bursts of thrombin generation at rates reported to be up to 10 times higher than those observed in normal pregnancy (7). The clinical implications of this case are elaborated in the following discussion.

### 3.1 Emergency warning for non-hypertensive HELLP syndrome

Our patient did not exhibit typical preeclampsia, yet developed life-threatening DIC driven by microangiopathic hemolysis, as evidenced by marked LDH elevation, and a precipitous platelet decline. Clinical data showed that both peak LDH levels and the rate of platelet recovery were significantly correlated with the pace of coagulation deterioration, supporting the use of dynamic changes in these parameters as early warning indicators (6, 8). Approximately 14% of HELLP patients do not present with hypertension, and in 30% of cases isolated abdominal pain is the initial symptom, underscoring the need to move beyond a “blood pressure-dependent” diagnostic paradigm (8). In this subgroup, the prevalence of DIC ranges from 15 to 38%, with a markedly elevated risk of coagulation deterioration, warranting heightened vigilance for a microthrombosis-dominated pathology (9, 10). According to the ACOG guidelines, patients presenting with unexplained coagulation abnormalities in the postpartum period—regardless of blood pressure—should undergo initial evaluation for HELLP syndrome. Recommended tests include platelet count (with ambulatory monitoring), LDH, liver enzymes (AST/ALT), and serum bilirubin, with a peripheral blood smear for schistocytes serving as a complementary diagnostic tool (11).

### 3.2 Differential diagnosis

Amniotic fluid embolism (AFE) was initially considered in the differential diagnosis because of the acute onset of coagulopathy shortly after delivery. However, several key clinical features were inconsistent with AFE and instead more indicative of HELLP syndrome: (1) Absence of cardiorespiratory collapse: The patient maintained SpO₂ ≥ 97% throughout, without electrocardiographic abnormalities or acute pulmonary edema—the hallmark presenting features of AFE. (2) Tempo of thrombocytopenia: The platelet decline followed a gradual, stepwise pattern consistent with HELLP syndrome. This stands in sharp contrast to the precipitous decline seen in AFE, where platelet counts decrease at a median rate of 60 × 10^9^/L/h, compared with 20 × 10^9^/L/h in HELLP (6); (3) Evidence of microangiopathic hemolysis: Marked LDH elevation (>600 U/L) and schistocytes (≥1%) on peripheral smear are highly specific for the thrombotic microangiopathy of HELLP syndrome. In contrast, hemolysis in AFE is typically mild, primarily driven by hyperfibrinolysis, with schistocyte counts usually <0.5% (12). Taken together, the absence of cardiorespiratory involvement, the characteristic platelet trajectory, and the definitive laboratory evidence of microangiopathy strongly support HELLP syndrome as the primary diagnosis.

### 3.3 Fibrinogen depletion

In this case, 3,000 mL of fluid was infused within 2 h postpartum, following the conventional volume expansion strategy for traumatic coagulopathy. However, this protocol may have aggravated the pathological cascade of HELLP-related DIC, through the following mechanisms: (1) Synergistic mechanisms of fibrinogen depletion: Although literature reports indicate that each 1,000 mL of crystalloid infusion typically reduces fibrinogen (FIB) by approximately 0.2–0.4 g/L (13), the magnitude of FIB decline in this case far exceeded what could be explained by dilution alone. This suggests that additional mechanisms were superimposed, including: ① coagulation factor consumption: in DIC, widespread microthrombus formation drives substantial fibrinogen depletion (14); ② hyperfibrinolysis: the patient’s markedly elevated D-dimer (>8.0 μg/mL) indicates secondary fibrinolytic activation, which further accelerates fibrinogen degradation. Therefore, the decline in FIB likely reflected the combined effects of dilution, consumption, and fibrinolysis. (2) Potential contributions of inflammation and endothelial injury: Excessive crystalloid infusion may exacerbate endothelial leakage and promote the release of inflammatory mediators through elevated hydrostatic pressure (15). In this case, the markedly elevated neutrophil ratio (94.9%) suggests a systemic inflammatory response, which may further disrupt the coagulation-fibrinolysis balance (16).

### 3.4 Acute kidney injury

The severity of the patient’s AKI was disproportionate to the degree of blood loss, as evidenced by the rapid rise in creatinine from 55 to 161 μmol/L over a short interval, suggesting that renal injury was mediated by multiple mechanisms: (1) Microthrombus embolism: Experimental studies demonstrate that HELLP syndrome complicated by acute kidney injury (AKI) significantly increases renal fibrosis, reduces glomerular filtration rate (GFR), and elevates urinary kidney injury markers (such as KIM-1, NGAL) (17), indicating that microthrombus formation and endothelial injury may dominate the renal pathological process; (2) Hemoglobin toxicity: Free hemoglobin can induce tubular ferroptosis through CD163 receptor-mediated pathways (16); (3) Imbalance of renal perfusion: Despite maintenance of mean arterial pressure (MAP) above 65 mmHg, microcirculatory disorders led to inadequate effective glomerular filtration pressure. This pathophysiological imbalance explains why high-dose furosemide failed to reverse oliguria in this case.

### 3.5 Clinical implications

In AKI associated with thrombotic microangiopathy (TMA), CRRT has been shown to remove inflammatory mediators and free hemoglobin, thereby mitigating renal injury (18). In cases of obstetric coagulopathy complicated by AKI, early initiation of CRRT is significantly associated with a reduced incidence of multiple organ failure (19).

## 4 Conclusion

This study has several limitations. First, the findings are derived from a single case report, which restricts the generalizability of the conclusions. Second, long-term follow-up data were unavailable because the patient did not undergo structured outpatient monitoring after discharge, precluding evaluation of long-term renal outcomes. In conclusion, this case of HELLP syndrome complicated by DIC without hypertension highlights a distinctive pathological mechanism in which microthrombus formation surpasses overt bleeding. The predominance of microvascular thrombosis observed in this case is consistent with emerging insights into immune-mediated coagulopathies. Recent evidence indicates that anti-β2-glycoprotein I-induced neutrophil extracellular traps (NETs) activate endothelial cells and upregulate tissue factor expression, thereby promoting a prothrombotic phenotype. This mechanism may underlie the rapid consumption of coagulation factors observed in our patient, even in the absence of hypertensive features (20). These findings underscore the need for a management approach distinct from conventional obstetric DIC models that focus primarily on “massive hemorrhage.” The dynamic relationship between microangiopathic hemolysis and abrupt platelet decline may serve as an early warning indicator of atypical HELLP. The heterogeneous mechanisms underlying thrombotic DIC warrant further investigation using microcirculatory monitoring techniques such as thromboelastography. Timely recognition and targeted intervention are essential to optimize patient outcomes and minimize the risk of severe complications. Future multicenter studies are needed to validate individualized coagulation management strategies and refine guideline recommendations.

## Data Availability

The original contributions presented in the study are included in the article/Supplementary material, further inquiries can be directed to the corresponding author.
